# Commentary: The physician as person framework: How human nature impacts empathy, depression, burnout, and the practice of medicine

**Published:** 2017-12-15

**Authors:** J Damon Dagnone

**Affiliations:** 1 Department of Emergency Medicine, Queen’s University, Ontario, Canada

In this issue of CMEJ, Lester Liao presents a theoretical framework with five key components of the physician as person and advocates for additional training opportunities for the integration of more humanities training within medicine.^1^ Let me comment as a physician whose life and professional identity have been forever and unfathomably transformed through and by a tragic event in my life. He contends that not only is separating out our own personal life experiences not possible in the practice of medicine, but is entirely inappropriate. I agree. Authentic and personalized interactions are an essential piece in caring for patients, whereby, the interrelatedness of life experiences should be at the centre of relationship building and meaningful connection for patients and physicians. In particular, evidence^2^,^3^ that suggests depression, burnout, and declines in empathy are on the rise demand that we explore new perspectives of understanding. In this article, the physician as person is more fully recognized and core principles are put forward to change the way we approach training physicians in the humanities, which has implications for us all.

The first principle of the physician as person framework states that encounters with patients occur primarily between two people. It is only when we as physicians put ourselves in “our patient’s shoes” that we can better connect with them on a more human level. This resonates with myself and many of my colleagues, as many of us have become much better, kinder, and more caring physicians as a result of our own healthcare experiences. Although medical training teaches us how to meet the needs of our patients, we actually learn more from our experiences of life and must leverage our own encounters to enhance the physician-patient dynamic. My defining and life changing moment came when my three-year-old son died from cancer. Today, not one shift goes by that my tragic life experience hasn’t affected at least one patient encounter on a daily basis. I am a different doctor having experienced the extreme challenges of living in hospital with a critically ill child, and, following his death, continuing to struggle with the hole that is forever in my heart. Listening to patients’ personal stories of their illness, I often find myself sitting on the side of their ER bed trying to make a more human connection with the person (the patient) beside me.

The second principle of the framework speaks to the interconnection of our personal and professional lives and states that we should avoid compartmentalization. As physicians, it is unhealthy to be a different person in the professional context than who we are in the personal context. It is also not natural or wholesome, and yet it can be frequently found as an insidious and powerful part of physician culture. Listening to patients, taking a history, performing a physical exam, embarking on a management plan, and communicating the essential parts of a clinical encounter must not be a mechanistic or strictly professional interaction that is separated from ourselves as people. To do these tasks effectively, a humanistic approach that makes a connection with the person (the patient) in front of you is what’s needed, and in my opinion, highly valuable.

The fourth of the five principles exposes the aspect of our culture that hides vulnerability and reinforces fictitious flawlessness. Each of us as physicians are individually imperfect in the care that we provide. This has always been the case, long before the added pressures of today’s overcrowded and overburdened healthcare system put us all at increased risk of error and overlooking or ignoring the individual needs of patients. Whether it is due to errors in our own diagnostic judgment, lack of access to resources, therapeutics errors, inappropriately caring for patients in hallways or corridors, and however big or little, it is an impossible standard to uphold flawlessness – and yet we too often try.

It is difficult to speak to flawlessness, and not consider vulnerability. The power of being vulnerable, of opening up and letting patients and colleagues see your imperfect humanity – apologizing, having tears, and sharing moments of pain – cannot be overstated and further reinforces the physician as person. It is no coincidence that humanity and humility share the same Latin roots. Yes, it is our duty as physicians to care for our patients and focus on their needs, but somehow in that commitment, we run the risk of losing out on being able to communicate our uncertainties, disappointments, failures, and challenges. As a bereaved parent, looking back at my experiences in hospital years ago, the most powerful connections I made with certain doctors was in large part due to their ability to be vulnerable with my wife and me. Towards the end of my son’s life, they did not have any answers and made some mistakes, and because they were vulnerable with us, we shared the burden of the difficulties together.

To me, there is no other way to approach caring for patients than to leverage the human interconnectedness in the patient-physician interaction. I applaud Lester Liao for his contribution of a new physician as person framework and I wonder if this could be the start of a discussion for an 8^th^ CanMEDS role or the addition of roots and a stem!
